# Treatment of anxiety disorders in clinical practice: a critical overview of recent systematic evidence

**DOI:** 10.6061/clinics/2019/e1316

**Published:** 2019-11-04

**Authors:** Vitor Iglesias Mangolini, Laura Helena Andrade, Francisco Lotufo-Neto, Yuan-Pang Wang

**Affiliations:** I Faculdade de Medicina FMUSP, Universidade de Sao Paulo, Sao Paulo, SP, BR; II Departamento de Psiquiatria, Instituto de Psiquiatria, LIM-23, Hospital das Clinicas HCFMUSP, Faculdade de Medicina, Universidade de Sao Paulo, Sao Paulo, SP, BR

**Keywords:** Anxiety Disorders, Therapeutics, Psychotherapy, Psychopharmacology, Systematic Review

## Abstract

The aim of this study was to review emerging evidence of novel treatments for anxiety disorders. We searched PubMed and EMBASE for evidence-based therapeutic alternatives for anxiety disorders in adults, covering the past five years. Eligible articles were systematic reviews (with or without meta-analysis), which evaluated treatment effectiveness of either nonbiological or biological interventions for anxiety disorders. Retrieved articles were summarized as an overview. We assessed methods, quality of evidence, and risk of bias of the articles. Nineteen systematic reviews provided information on almost 88 thousand participants, distributed across 811 clinical trials. Regarding the interventions, 11 reviews investigated psychological or nonbiological treatments; 5, pharmacological or biological; and 3, more than one type of active intervention. Computer-delivered psychological interventions were helpful for treating anxiety of low-to-moderate intensity, but the therapist-oriented approaches had greater results. Recommendations for regular exercise, mindfulness, yoga, and safety behaviors were applicable to anxiety. Transcranial magnetic stimulation, medication augmentation, and new pharmacological agents (vortioxetine) presented inconclusive benefits in patients with anxiety disorders who presented partial responses or refractoriness to standard treatment. New treatment options for anxiety disorders should only be provided to the community after a thorough examination of their efficacy.

## INTRODUCTION

According to the World Health Organization (1), anxiety disorders are burdensome “common mental disorders” to communities. These prevalent disorders are not communicable and affect approximately one in every five individuals of the world population (2-4). This figure represents the largest share of the prevalence of all mental disorders, whereas severe psychotic and bipolar disorders affect only between 1% and 2% of the population. In an upper-middle income country such as Brazil, the 12-month prevalence of anxiety disorders has been estimated as 19.9% among the dwellers of a large metropolitan area (5).

The cost of anxiety disorders to the working world is remarkable, corresponding to a total loss of 74.4 billion Euros in 2010 (3). The global burden of anxiety disorders represents 10.4% of years lived with adjusted disability (DALY) of mental disorders, reaching 26,800,000 DALYs (2). Despite the societal burden of this morbidity, only approximately one in five patients diagnosed with anxiety disorder obtain access to treatment (6,7).

**Figure 1:**
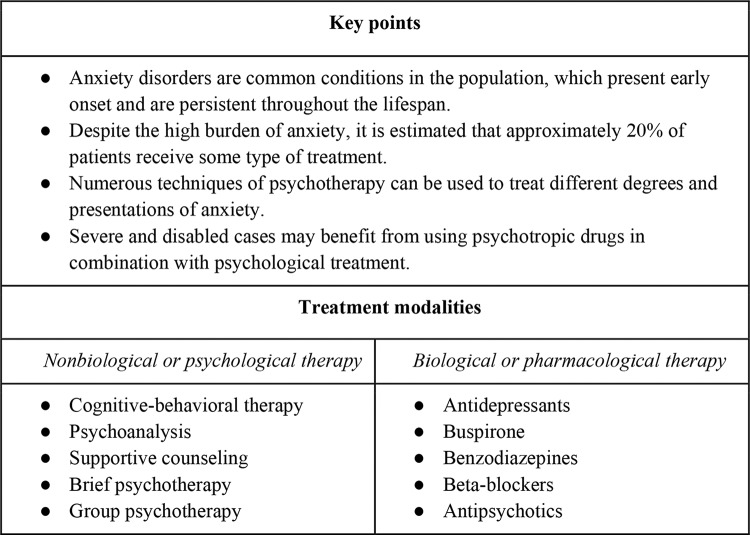
What we already know about the treatment of anxiety disorders (9,10,11).

Anxiety disorders present an early onset, even during childhood. Their enduring waxing and waning course deeply affects patients’ functionality and interpersonal relationships throughout the lifespan (8). Most pathological anxiety (specific phobias, social anxiety, generalized anxiety, separation anxiety, obsessive-compulsive, and panic disorder) is underrecognized, and patients seek treatment in outpatient settings, either in medical or specialized mental health-care contexts (7). However, anxiety disorders receive less attention from clinicians when compared with major mental disorders, such as psychotic conditions and substance use disorders that require hospitalization. Moreover, anxiety is less reported in the media than depression and suicide attempts, which reduces the help-seeking behaviors of patients suffering from anxiety. Figure 1 summarizes key uncontroversial characteristics and clinical practices regarding the treatment of anxiety disorders (9-11). Most experts advocate either psychotherapy and/or pharmacotherapy for alleviating or controlling symptoms of anxiety. The combination of psychological treatment with psychotropic drugs is recommended for patients with severe cases of disabling anxiety.

Traditionally, several talk therapies are subsumed as techniques of psychological treatment and have been recommended to handle different degrees of anxiety (11). Well-accepted but not always efficacious modalities of psychotherapy vary from psychoanalytic, cognitive-behavioral, interpersonal, supportive, and group therapy to brief therapy. The literature on cognitive-behavioral therapy (CBT) has established a foundation of effectiveness evidence for different anxiety disorders (9,11), but new therapeutic modalities should have their benefit assessed. In addition, the existing number of mental health professionals is insufficient for the number of patients who need treatment (6). Thus, a more accessible and cost-effective modality of psychotherapeutic treatment for anxiety should be offered to the community.

More than six decades ago, since the synthesis of chlordiazepoxide in 1957 (12), benzodiazepine medications have become the main class of pharmacological agents for the treatment of anxiety disorders. The introduction of these anxiolytic medicines received an immediate welcome from medical professionals and anxiety-laden patients. Nonetheless, the risk of side effects, a withdrawal syndrome and dependence on benzodiazepines have led patients in need of treatment to seek less harmful therapeutic substitutes, which do not always have proven efficacy. Accepted psychopharmacological medicines include antidepressants, buspirone, beta-blockers, and antipsychotics. Their efficacy has been demonstrated in well-designed clinical trials and abridged in comprehensive reviews (10). The combined use of psychological treatment with psychotropic drugs is more commonly recommended for cases of anxiety of greater severity and disability (11).

Many complementary and alternative treatments of mild forms of anxiety have gained popularity because of their alleged harmlessness. Examples of complementary treatment include aromatherapy, acupuncture, herbal medicine, homeopathy, massage therapy, yoga, mindfulness, exercise practice, relaxation, etc. (6,7). The diversity of modalities that a patient is exposed to varies in accordance with the guidance of the therapist, use of an active substance, and body manipulation. Exhaustive classification is difficult. While mental health professionals support the adjunctive addition of these modalities, for anxiety disorders in particular, the exclusive use of alternative therapies as a surrogate to well-established forms of treatment should be avoided (11). Most complementary and alternative treatments lack evidence of effectiveness. It is possible that a placebo effect and a good therapeutic relationship between the practitioner and patients underlie their positive outcomes.

There are a wealth of treatments devoted to controlling the symptoms of anxiety, but nonconventional and newer psychotherapeutic treatments and pharmacological agents are propagated without an acceptable confirmation of benefit. In the present review, we searched for recent evidence of nonbiological (psychological) and biological (pharmacological) modalities for treating anxiety disorders. The comprehensive summary of treatment advances is organized for a professional who is in training or is not a specialist in mental health to supplement existing modalities. Complementary and alternative treatments with evidence of effectiveness are explored herein under the group of nonbiological therapies. Additionally, high-quality systematic reviews (SRs) were chosen over sparse clinical trials in need of additional replication. The usefulness and public health importance of the treatment of anxiety are subsequently discussed.

## METHODS

Our research question was to update the evidence on recent interventions for the broad category of anxiety disorders. In the present study, the PICO components included adult Patients with a clinical diagnosis of “anxiety disorder”, who were subjected to one or more Interventions (either biological or nonbiological). The intervention must be Compared with a placebo or standard therapeutics for assessing the treatment Outcomes.

We searched for articles in the PubMed and EMBASE databases on the treatment of anxiety disorders. The key Medical Subject Heading (MeSH) terms were “anxiety disorders” AND “treatment”. The retrieved articles were displayed in the Mendeley platform and filtered in accordance with the Preferred Reporting Items for Systematic Reviews and Meta-analysis (PRISMA) guidelines (13). The arguments of the search strategy can be found in Supplementary Table 1.

For inclusion, the article type must be an SR, with or without meta-analysis, of clinical trials involving adult patients diagnosed with an anxiety disorder. Rigorous randomized clinical trials (RCTs) compared with placebo or active interventions were considered the highest evidence of effectiveness. Those articles wherein participants encompassed a mixed sample of adults and children were not eligible unless separate data were comprehensively presented. Only articles published in the last 5 years, from January 2013 through September 31, 2018, were considered appropriate. There was no language restriction regarding published articles.

After hand searching, by reading the reference list of retained articles and chapters, and contact with potential authors, we identified two additional articles (14,15).

Regarding exclusion criteria, articles containing primary data, duplicate SR or animal models of anxiety were not eligible. Posttraumatic stress disorder was not considered in the present overview because this disorder is not covered under the MeSH term “anxiety disorders” and is no longer listed in the DSM-5 chapter of anxiety disorders (16). In contrast, while the DSM-5 describes obsessive-compulsive disorders in a separate chapter, this group of disorders is still listed under the MeSH entry of anxiety disorders. Furthermore, treatments on the cooccurrence of anxiety disorders in a specialized medical context (e.g., heart disease, endocrinological, neurological conditions, pain clinics, etc.) were eliminated. Observational studies, case reports, comments, practice guidelines and editorials on therapeutic modalities were also excluded from this overview. Two authors (V.I.M. and Y.P.W.) decided the final list of selected articles.

### Study method

Often, an individual SR cannot address all proposed interventions for the same problem. Recent advances in the treatment of anxiety disorders are updated in the current study with the methodological framework of a systematic overview (17). Accordingly, this type of meta-review is a relatively new method to achieve a high level of evidence, wherein systematic evidence gathered from more than one SR or meta-analysis is examined in a single accessible work, also known as a “systematic review of systematic reviews” (17). The compilation of evidence synthesizes different interventions for the same problem or condition on different outcomes for different conditions, problems or populations. The ultimate result provides a global summary of the available evidence rather than providing data synthesis (17,18). Thus, an overview aims to examine the highest level of evidence and provide a global account of findings (19). This type of review has the advantage of rapidly combining relevant data to make evidence-based clinical decisions. Stakeholders, managers and health professionals can appraise multiple high-quality studies in a single general summary of a particular question.

The quality of the retained review articles was assessed in accordance with “A MeaSurement Tool to Assess systematic Reviews” (AMSTAR version 2) (20). The 16-item AMSTAR checklist (https://amstar.ca) represents a critical appraisal of the quality of SRs, covering different aspects related to study planning and conduct, such as the research question, review protocol, selection of study design, search strategy, explicit inclusion and exclusion criteria, risk assessment of bias, and publication bias. For the interpretation of detected weaknesses in critical and noncritical items, the AMSTAR recommends a categorization of the overall confidence in the results of the SR as follows: high, moderate, low, and critically low. The assessment of the risk of bias of an SR was supplemented with the Risk Of Bias In Systematic review (ROBIS) guidelines (21), which allows classification of the existence of bias as low, high or unclear. All rating disagreements were reconciled during discussion meetings.

## RESULTS

**Figure 2:**
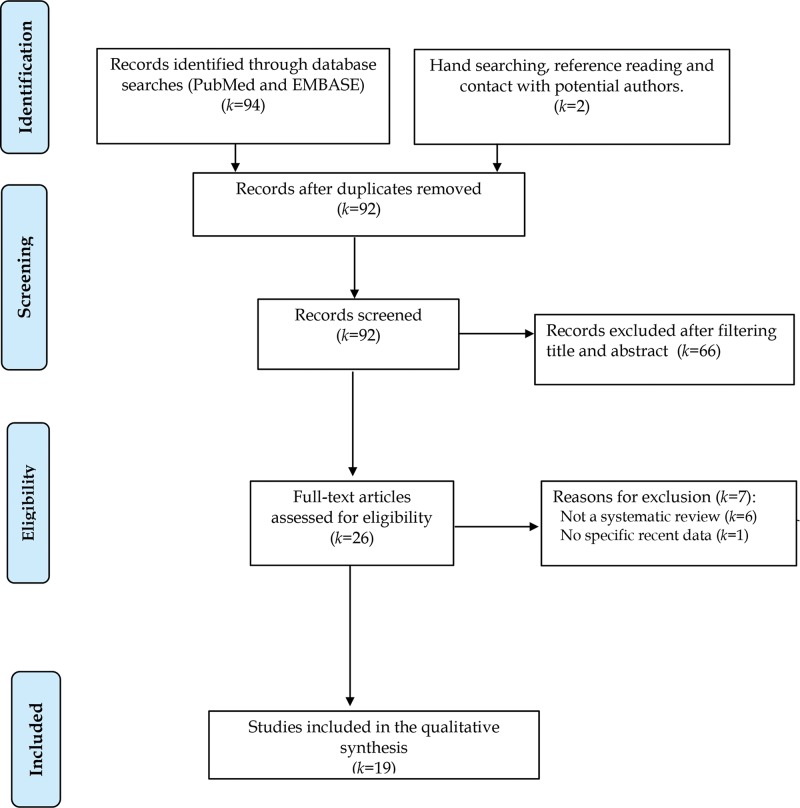
Flow diagram according to PRISMA (http://www.prisma-statement.org) for identifying eligible articles (*k*=number of studies).

Figure 2 shows the PRISMA flow diagram of the retrieved articles in this overview. From the initial 96 review articles published between 2013 and 2018, 92 nonduplicated articles were screened for title and abstract. Most studies (*k*=66) were removed because the participants presented anxiety symptoms in the context of medical diseases or were nonadults. After eliminating ineligible articles that fell outside the topic of overview, 26 articles were retained for full-text reading. An additional 7 articles were excluded because 6 did not present an SR and 1 did not contain recent data. The reasons for article exclusion can be found in Supplementary Table 2. Accordingly, 19 recent SRs were included in the final list for the qualitative synthesis. Of these studies, 3 did not estimate the pooled effect size of the outcomes through a meta-analytical quantitative synthesis (22-24).

Table 1 summarizes the main characteristics and methods of the 19 retained studies. From these articles, 11 referred to nonbiological treatments for anxiety (media- or internet-assisted CBT therapy, brief psychodynamic therapy, Morita therapy, effects of safety behavior, practices of exercise, mindfulness, and yoga, etc.), 5 referred to biological treatments for anxiety (repetitive transcranial magnetic stimulation and pharmacotherapy), and 3 referred to multimodal combined treatment comparisons (stepped care *vs*. care-as-usual and comparison of multiple treatments). All articles were published in English, and the investigators had searched for relevant articles in at least two databases. Although our search was restricted between 2013 and 2018, the majority of retained SRs covered the previous period, from the database inception date up to 2017.

Across the SRs, there were a total of 811 RCTs (range: 2–234 RCTs), with an included total of 87,773 adult participants (range: 40-37,333 patients). Three SRs (15,35,36) included over 10,000 participants, 6 SRs (25-29,37) between 9,999 and 1,000 participants, 8 SRs less than 1,000 participants (22,23,30-34,38), and 2 SRs did not report the exact number due to the mixture of adult and underage participants (14,24). Most SRs (*k*=14) did not report or summarize the percentage of female participants. The other 5 SRs (25,28,30,33,38) indicated the proportion of women (range: 55.5%-67.7%).

Regarding the diagnosis of the participants, the majority of studies investigated the disorder either under a generic diagnostic label of anxiety disorders or common mental disorders. SRs evaluated the effects of specific interventions in social anxiety (14,15,23,24,35), panic (14,15,33), generalized anxiety (14,15), and obsessive-compulsive disorder (36). All articles described the exclusion of ineligible participants (e.g., posttraumatic stress or acute stress disorders, depressive disorders, comorbid physical illnesses, psychotic disorders, nonappropriate psychiatric diagnoses, underage participants, etc.) and inappropriate studies (e.g., small sample size or case studies, sampling or statistical issues, unsuitable interventions, etc.).

The Cochrane’s Collaboration Tool to Assess Risk of Bias was the most commonly used instrument (*k*=14) to evaluate the risk of bias in each individual SR. Two SRs (14,15) used the Scottish Intercollegiate Guidelines Network (SIGN) checklist, and an additional 3 SRs (24,36,37) did not assess the risk of bias.

### Evidence of treatment efficacy

Regarding the results of nonbiological or psychological treatments, 5 SRs evaluated computer-delivered psychological therapy (14,15,25,26,28). The evidence suggested that the online therapeutic approach is a feasible and beneficial treatment option. However, face-to-face therapist-guided therapy seemed to be clinically superior when compared with the computer-guided approach. Additionally, the benefit widely varied in accordance with the type and characteristics of anxiety disorder.

A meta-analysis (27) reported that short-term psychodynamic psychotherapies appear to show a reduction in anxiety symptoms in the short and medium term. The SR of Morita therapy-a specific type of self-acceptance method-showed data of limited applicability because all eligible studies were conducted in China, restricting the utility of conclusions in Western countries (30).

Three SRs (23,24,35) had specifically included patients with social anxiety. Mindfulness and acceptance-based treatment (23) was a viable option, but the level of evidence was limited due to the risk of bias. For social anxiety, limited evidence suggested that reductions in the use of safety behaviors or avoidance were related to a better CBT outcome (24). In addition, symptomatic decreases in social anxiety predicted reduced safety-behavior use over the course of treatment.

Two SRs (22,31) evaluated the benefit of exercise in reducing anxiety symptoms. Both studies indicated that the exercise practice was effective, regardless of the type and intensity of physical activity. However, exercise alone was less effective than standard antidepressant treatment (15). Although the effect of yoga on anxiety disorder was considered a safe intervention, the gathered evidence for its effects was inconclusive (32). Main critiques referred to the variety of diagnoses, heterogeneity of interventions, potential bias of low-quality studies, and lack of comparison to other treatments.

Regarding biological or pharmacological treatments, one meta-analysis (33) assessed transcranial magnetic stimulation in 40 participants with panic disorder. However, there was insufficient evidence to draw any solid conclusion about its efficacy because of the small sample size and significant methodological flaws. In addition to sampling issues (randomization and allocation concealment), the evidence in the 2 RCTs reviewed was of very low quality.

For pharmacological treatments, there was evidence of low-to-moderate quality for the use of selective serotonin reuptake inhibitors (SSRIs) for social anxiety (35). However, their tolerability seemed to be lower than placebo. The augmentation strategy did not appear to be beneficial in patients with treatment-resistant anxiety disorders, e.g., generalized anxiety, social anxiety, and panic disorder (34). In a comparison of the effects of second-generation antidepressants for obsessive-compulsive *vs*. generalized anxiety disorder, panic disorder, posttraumatic stress disorder, and social anxiety disorder (in over 15,000 participants), an SR (36) found that pharmacotherapy presented a smaller overall change score than placebo for those five categories of anxiety disorders. Finally, an SR of incipient trials of vortioxetine supported its use for anxiety (37), but more long-term placebo-controlled trials are warranted.

The SR on multimodal combined treatments reviewed 10 RCTs and compared the package of stepped care *versus* care-as-usual (38). The authors concluded that the stepped-care model of treatment of anxiety disorders appeared to be superior than care-as-usual in terms of efficacy and cost-effectiveness. As a consequence, stepped care can reduce the burden on service providers and increase availability. In a comprehensive SR on multiple treatment modalities with over 37 thousand participants (15), the average pre-post effect sizes of medications were more effective than psychotherapies. In general, the effects of psychotherapies did not differ from placebo pills. Surprisingly, not only psychotherapy but also medications and, to a lesser extent, placebo conditions have shown similar enduring effects in the improvement of anxiety disorders (14). Nevertheless, long-lasting treatment effects observed in the follow-up period were superimposed in patients receiving different therapeutics at the same time.

### Quality of evidence

Using the AMSTAR guideline, Table 2 presents the assessment of the quality of each individual SR. The overall confidence of each study was rated after evaluating critical and noncritical items of the AMSTAR. Several SRs (*k*=6) were rated as high quality (25,27,28,30,33,35); 3, as moderate (23,26,31); 7, as low (14,15,22,29,31,34,38); and 3, as critically low (24,36,37). All six reliable articles (AMSTAR high quality and ROBIS low risk of bias) were published in the Cochrane Database of Systematic Reviews and rigorously adhered to the guidelines of the Cochrane’s Collaboration Tool to Assess Risk of Bias.

Most of the studies clearly described the planning phase of the SR, which included explicit research questions, selection criteria, data extraction and assessment of the risk of bias. Not all studies previously registered a protocol before performing the SR. Only 3 studies reported the source of funding of the included studies (25,30,35). During the data interpretation, the most frequent problems were no clear discussion of the individual bias of selected studies (*k*=9) and did not account for publication bias (*k*=5). Notably, the 3 SRs that did not subject the RCTs to a meta-analytical synthesis also presented several shortcomings that critically affected the quality of the articles (e.g., omission of excluded studies, nonevidence-based discussion of results, and no prior protocol registration).

The risk of bias was rated with the aid of ROBIS (Table 2), with 8 SRs having low risk (25-28,30,31,33,35); 8, uncertain risk (14,15,22,23,29,31,34,38); and 3, high risk (24,36,37). There was a rough agreement between the quality of an SR (AMSTAR) and the risk of bias (ROBIS). Unsurprisingly, while most high-to-moderate quality studies presented a low risk of bias, all three studies of critically low quality also presented a high risk of bias (24,36,37). In Supplementary Table 3, detailed ROBIS ratings for each retained study are shown.

## DISCUSSION

The current overview summarized the evidence of the efficacy of emerging treatment options in the last 5 years for adult patients with an anxiety disorder. The conclusions of 19 relevant SRs were synthesized and combined, for a total of 87,773 participants distributed in 811 RCTs. There was great cross-study heterogeneity in terms of the research question, target disorder, type of intervention, methodology, number of included RCTs, sample size of participants, and measured outcomes. Most studies investigated the benefit of different forms of psychotherapy and physical activity. In terms of biological treatments, no great evidence of effectiveness was found for transcranial magnetic stimulation and pharmacological strategies (drug augmentation or novel agents).

Newer treatments for anxiety disorders are highly relevant because the majority of cases are underdetected and undertreated within health-care systems, even in economically developed countries (14). Most anxious patients worldwide do not receive standard treatment with combined psychotherapy and pharmacological agents in terms of adherence, frequency, and adequacy (6,9,11). Consequently, untreated patients with these disorders chronically endure these symptoms, which are associated with severe impairments and restrictions in role functioning and disabilities (6). The present overview of SRs presented a resynthesis of existing data to allow better choices among emerging interventions for anxiety disorders. This rapid review of high-quality evidence can be of great clinical utility for decision-makers and public health administrators. Until more robust evidence is published, the initial enthusiasm for many proposed anti-anxiety alternatives has shrunk. Meanwhile, the evidence of many therapeutic alternatives should be watchfully disseminated to the community.

### Interpretation and implications

From the present overview, there is convincing evidence that computer-delivered psychological treatment is helpful for the treatment of distressing anxiety of different intensities (25). However, the therapist-oriented CBT approach has yielded better results (25,28). Along similar lines, short-term psychodynamic psychotherapies have shown consistent gains, but larger studies with specific anxiety disorders are warranted (27). From a public health standpoint, computer-assisted treatment is not readily accessible in several nondeveloped countries, but this strategy can benefit those patients living in distant places or unwilling to start formal psychotherapy. Furthermore, sharing a single computer device and delivering brief psychotherapy are cost-effective for a community (40).

There is evidence of moderate-to-high quality suggesting that the online approach may be favorable and more efficacious than a wait list, informational pamphlets, or online discussion groups (25). Therefore, the self-help approach can be recommended as the first step in the treatment of mild anxiety disorders, but the short- and long-term effects of computer-delivered interventions and brief psychotherapies need to be fully established.

Although the SR of Morita therapy was of high quality and free of the risk of bias, its applicability is limited (30). All 7 RCTs of Morita therapy were conducted in Eastern countries, curbing its generalizability to Western populations (41).

Two promising high-quality SRs still required additional evidence of effectiveness with additional RCTs; pioneering transcranial magnetic stimulation (33) and the use of SSRIs in social anxiety (35) have shown insufficient evidence of efficacy. The SR of transcranial stimulation studies was conducted on 2 RCTs with 40 patients with panic disorder. Therefore, further trials with a larger sample are needed. The use of SSRIs in social anxiety has shown low-to-moderate evidence of efficacy and was less tolerable than placebo (35). These two strategies can be advised for specific anxiety disorders and those patients who presented partial response or refractoriness to standard treatment (35,42-45). In a further meta-analysis based on weekly outcome data (46), the treatment benefits of SSRIs and serotonin norepinephrine reuptake inhibitors (SNRIs) were shown for social anxiety. Higher doses of SSRIs, but not SNRIs, were associated with symptomatic improvement and treatment response. However, the potential risk of intolerance may surpass the benefit to the patients (46).

With an ever-growing list of psychotropic compounds showing apparent anxiolytic properties, current pharmacological options for treating clinical anxiety are broad and vast. Existing SRs (14,15) demonstrate that the magnitude of efficacy for most anxiolytic agents compared with placebo was superior. However, the likelihood of symptomatic remission after a pharmacological trial remains largely unknown. Progress in neuroscience and neurophysiology may unravel the pathways of therapeutic responsiveness.

Thus, the generalizability of emerging treatments, e.g., transcranial stimulation and newer pharmacological strategies, is limited due to sampling issues, methodological flaws, and applicability in specific anxiety disorders. These potential interventions might not be available to all consumers, and therefore, larger and more pragmatic RCTs are needed to evaluate and maximize the benefits of available interventions (42-45).

Behavioral recommendations of regular exercise (22,31), mindfulness practice (23), and yoga (32) have also been shown to be beneficial for improving anxiety symptoms. However, these SRs were of low-to-moderate quality and vulnerable to the risk of bias. The universal campaign of healthy activities might be recommended as an adjunctive treatment to standard treatment and a cost-effective strategy in regions where there is a shortage of qualified therapists. Nonetheless, these practices were less effective when compared with antidepressant pharmacotherapy (15). Even without sufficient evidence of effectiveness, these nonstandard treatments seem to be safe, inexpensive and can be easily implemented with preventive purposes to community dwellers (47).

Although methodological questions remain before its broad implementation can be supported, the personalized therapist-guided CBT approach is the most recommended nonpharmacological treatment for anxiety (48). Similarly, while the practice of physical activities is safe and helpful, traditional antidepressant treatment presents better results (9,14). One unanswered question refers to the potential adverse effects of the nonsupervised use of computer-assisted therapies and exercise practice. These concerns need to be refined in future RCTs.

Among those patients receiving long-term treatments with partial response or refractoriness, it is possible that novel strategies can enhance and sustain the improvements in anxiety. Hence, there is a large amount of room for amendments to treatment plans (34-38), at least for specific and severe anxiety disorders. Future studies should include stratification of anxiety by severity status and persistence to characterize the dose-response relationship of interventions and the combined efficacy of psychotherapy and pharmacotherapy in treating anxiety disorders, in addition to rule out potential confounding factors that affect treatment effectiveness (49,50).

Some SRs were untrustworthy due to their low quality and serious biases. For example, the impact of safety behaviors in social anxiety remains unknown (24), as well as the reduced response to placebo and antidepressants in obsessive-compulsive disorders (36) and the benefit of vortioxetine for the treatment of anxiety disorders (37). In general, the most common shortcomings were the lack of a published protocol, unclear study selection, inadequate search strategy, lack of explicit inclusion and exclusion criteria, nonexhaustive assessment of bias, invalid interpretation, and no report of publication bias. Consequently, these topics require urgent clarification, using a more stringent methodology and longer follow-up to answer the proposed research question.

### Limitations

The heterogeneous interventions reported in these SRs with diverse outcomes preclude conducting a quantitative meta-analytical synthesis as an umbrella review (17-19,39). However, the present systematic overview has assessed the risk of bias of each individual SR, and it is secure to claim that most of the evidence reported herein was trustworthy.

The search for recent SRs on the treatment of anxiety disorders has identified main review articles, but some gray literature might have been missed. Although the studies in the Cochrane library were covered in PubMed and EMBASE, ongoing SRs must be finalized to draw solid conclusions. Along these lines, the Cochrane register and PROSPERO data were not scanned to detect other SRs. However, preliminary findings or unpublished SRs should not be integrated into the present overview. It is possibly that a selection bias of new treatment alternatives for specific anxiety disorders occurred at the time of the search. The potential omission of ongoing RCTs cannot be ruled out, but untrustworthy or partial evidence should not be taken as high-quality information.

A potential bias of overview studies is overlap in the retrieved articles or the use of the same primary study in multiple included SRs (51,52). In the present review, most of the treatment modalities were addressed by only one included SR, which probably reduced the probability of overlap across those studies. However, there were two interventions that were addressed by multiple studies: media-delivered psychotherapy and physical exercises. Five SRs examined media-delivered psychotherapy, with a total of 463 RCTs included in the reviews. It is possible that overlap occurred across these SRs, and subtle differences exist regarding the sample, scientific question, comparator, and inclusion of therapist. Therefore, we cannot rule out the possibility of overlapping articles, and the strength of the conclusion about media-delivered psychotherapy should be softened. In contrast, in the two existing SRs on physical exercises, we found 16.7% overlap across the included RCTs. In addition, the overall quality of the articles on physical exercise was low-to-moderate according to the AMSTAR analysis. This fact likely endorses the lower efficacy of physical exercises than standard care.

The covered period of five years may have not included all published studies before 2013. Nevertheless, these recent articles have offered updated coverage of previous studies conducted more than five years ago. Because our primary goal was to condense recent advances on the evidence-based therapeutics for anxiety, well-known modalities were outside the scope of the present review. Notwithstanding, two comprehensive meta-analyses conducted by Bandelow’s group (14,15) provided a broad summary of existing evidence on treatments for anxiety disorders, as well as the comparative enduring effect of psychological treatments and efficacy of treatments.

Trials with negative results might remain unpublished, and practitioners continue advising off-label use without any evidence of effectiveness or benefit. This publication bias of the file drawer effect cannot be ruled out. Small study bias and excluded participants may have affected the scientific soundness of the conclusions. For example, repetitive transcranial stimulation still requires a larger sample (42-45), and Morita therapy should be investigated in Western countries and regions in different stages of development (41).

## CONCLUSIONS

The present overview of recent treatment trends for anxiety disorders provides an account of the evolving directions to pursue, in terms of state-of-art scientific development. Effective and older treatments should be enhanced with technological innovations such as computer-based CBT and supplemented by adjunctive physical activities. New biological or pharmacological treatment modalities for anxiety disorders still need further evidence of usefulness. Thus, all treatments for anxiety disorders with proven effectiveness should be continuously investigated to make them available to the community.

The worldwide burden of anxiety disorders is high. Therefore, obtaining access to reliable health-care services is a bonafide and essential need in a globalized world. However, direct-to-consumer universal access to emerging treatments for anxiety should be recommended only after demonstration of robust evidence of efficacy.

## APPENDIX

**Supplementary** **Table 1** **-** Search Strategies 

SEARCH

DATABASE #1

**PubMed**

- Article types: Review
- Time period covered: Last 5 years
- Language: English, Portuguese and Spanish
- Age: Adults 19+
- Species: Humans

Search strategy:

anxiety disorders[Title/Abstract] AND treatment[Title/Abstract] AND (Review[ptyp] AND “2013/01/01”[PDAT] : “2018/12/31”[PDAT] AND “humans”[MeSH Terms] AND (English[lang] OR Portuguese[lang] OR Spanish[lang]) AND “adult”[MeSH Terms])

*# of articles retrieved: 72*

DATABASE #2

**EMBASE**

- Article types: Review
- Time period covered: 2013-2018
- Language: English, Portuguese and Spanish
- Age: Adults
- Species: Humans

Search strategy:

‘anxiety disorder’:ab,ti AND ‘treatment’:ab,ti AND [review]/lim AND ([english]/lim OR [portuguese]/lim OR [spanish]/lim) AND [adult]/lim AND [humans]/lim AND [2013-2018]/py

*# of articles retrieved: 22*

## AUTHOR CONTRIBUTIONS

Mangolini VI and Wang YP contributed equally to the manuscript and were responsible for the study conception, data acquisition and extraction, and manuscript drafting. Andrade LH and Lotufo-Neto F have critically reviewed the discussion and conclusion. All of the authors approved the final version of the submitted manuscript.

## Figures and Tables

**Table 1:** Characteristics of 19 systematic reviews on the treatment of anxiety disorders (2013-2018).

Author, Year	Research question	Period	Studies	Participants	*N*	Women	Interventions	Exclusion	Main Outcomes	Quality of evidence	Conclusions
**Nonbiological or psychological treatments**											
Mayo-Wilson, 2013 (25)	Media-delivered behavioral and cognitive behavioral therapies	Up to 2013	101 RCTs	Adults with anxiety disorders	8,403	67%	CBT and behavioral therapy, media-delivered alone or as adjuncts to another treatment	PTSD and acute stress disorder	Change in symptoms of anxiety: continuous symptom measures, response and recovery	Cochrane	Self-help may be useful for people who cannot use other services. However, face-to-face CBT is probably clinically superior.
Jayakody, 2014 (22)	Exercise vs. other treatments	Up to 2011	8 RCTs	Adults with anxiety disorders	563	NR	Different forms of exercise (alone or in combination with other treatments)	Depressive disorders	Changes in symptoms of anxiety, improvement in mental state or quality of life, relapse, and compliance with exercise treatment	Cochrane	Exercise seems to be effective as an adjunctive treatment, but it is less effective than antidepressant treatment.
Arnberg, 2014 (26)	Internet-delivered psychological treatment	Up to 2013	40 RCTs	Participants* with anxiety or mood disorders	2,622	NR	Theory-based psychological interventions, as delivered via the internet	Primary physical illness	Change in symptoms of anxiety, adverse events, and cost per effect and per quality-adjusted life-years	Cochrane	Internet-based CBT is a viable treatment option. Methodological questions remain before broad implementation can be supported.
Abbass, 2014 (27)	Efficacy of short-term psychodynamic psychotherapies	Up to 2014	33 RCTs	Adults with common mental disorders	2,173	NR	Individual short-term psychodynamic psychotherapies or approaches (40 weeks on average, 45- to 60-minute sessions)	Psychotic disorders	Improvement in general symptoms as measured by psychiatric instruments or criteria and somatic symptoms	Cochrane	Short-term psychodynamic psychotherapies show modest to large gains. Larger studies of higher quality and with specific diagnoses are warranted.
Norton, 2015 (23)	Mindfulness and acceptance-based treatment	Up to 2014	9 RCTs	Adults with social anxiety	330	NR	Mindfulness and acceptance-based treatment	No statistical analyses, irrelevant interventions, not peer reviewed studies	Changes in cognitive, behavioral, and physiological symptoms	Cochrane	The benefit of mindfulness and acceptance-based treatment can be considered a viable alternative. CBT remains best practice for first-line treatment of social anxiety.
Olthuis, 2015 (28)	Therapist-supported internet cognitive behavioral therapy	Up to 2015	38 RCTs	Adults with a primary anxiety disorder	3,214	67.7%	Therapist-supported CBT delivered via internet (web pages or e-mail)	Other comorbidity and anxiety symptoms that did not meet diagnosis criteria	Clinical improvement determined by interview and reduction in symptoms of anxiety by scores	Cochrane	Therapist-supported internet-based CBT appears to be an efficacious treatment for anxiety in adults.
Newby, 2015 (29)	Clinician-guided internet/computerized or face-to-face treatments	Up to 2014	50 RCTs	Adults with a primary anxiety or depressive disorder	1,865	NR	Manualized psychological treatments (at least 2 sessions)	Insufficient data, under age 18, case studies, and case series	Improvement in symptoms of anxiety, as measured by instruments and quality of life scores	Cochrane	Transdiagnostic psychological treatments are efficacious, but higher quality research studies are needed.
Wu, 2015 (30)	Morita therapy	Up to 2014	7 RCTs	Adults with anxiety disorders	449	55.5%	Morita therapy by the carers (at least two of the four phases)	Secondary anxiety symptoms of a different disorder, comorbid disorders	Clinical response, dropouts and measure of total acceptability.	Cochrane	The evidence base on Morita therapy was limited. All included studies were conducted in China, curbing the applicability of conclusions to Western countries.
Piccirillo, 2016 (24)	Safety behaviors in social anxiety	Up to 2015	39 RCTs	Adults with social anxiety	NR	NR	Exposure to safety behaviors as attempts to prevent or avoid feared outcomes (threatening or catastrophic) during CBT	No data on safety behaviors, children and adolescent, not in English, case studies, not social anxiety	Change in measures of safety behaviors, e.g., Social Behaviors Questionnaire (SBQ) and Subtle Avoidance Frequency Evaluation (SAFE)	NR	Limited evidence suggests that reductions in the use of safety behaviors are related to better CBT outcomes, and reductions in social anxiety predict reduced safety-behavior use over the course of treatment.
Stubbs, 2017 (31)	Exercise in people with anxiety and/or stress-related disorders	Up to 2015	6 RCTs	Adults with a primary anxiety or stress disorders	262	NR	Exercise vs. a nonactive group (usual-care, wait-list, placebo or social activities)	Yoga, tai chi or qigong; and comparison with active treatments (pharmacotherapy or psychotherapy).	Mean change in anxiety symptoms in the exercise vs. control group according to a validated outcome measure	Cochrane	Data suggest that exercise is an effective intervention in improving anxiety symptoms in people with anxiety and stress-related disorders
Cramer, 2018 (32)	Effectiveness of yoga	Up to 2016	6 RCTs	Adults with anxiety disorders	319	NR	Multicomponent yoga, posture-based yoga, and breathing/meditation-based yoga	Obsolete diagnoses	Improvement in severity of anxiety and remission	Cochrane	Yoga is effective and safe for individuals with elevated anxiety. There was inconclusive evidence for effects of yoga in anxiety disorders.
**Biological or pharmacological treatments**											
Li, 2014 (33)	Repetitive transcranial magnetic stimulation	Up to 2014	2 RCTs	Adults with panic disorder	40	60%	Repetitive transcranial magnetic stimulation of high or low frequency (alone or in combination with other interventions)	Single-pulse intervention, or treatment period of less than one week	Effectiveness measured by symptom severity, and acceptability: dropouts and adverse effects	Cochrane	There is insufficient evidence to draw any conclusions about efficacy. Further RCTs are needed.
Patterson, 2016 (34)	Augmentation strategies in treatment-resistant anxiety	1990-2015	6 RCTs	Treatment-resistant adults with anxiety disorders	557	NR	Pharmacotherapy or CBT augmentation of a first-line SSRI (with a placebo control)	Concomitant medication trials or not SSRIs as first-line treatment	Clinical Global Impression, changes in symptom severity, disability and functional impairment	Cochrane	Augmentation does not appear to be beneficial in treatment-resistant anxiety disorders
Williams, 2017 (35)	Pharmacotherapy for social anxiety disorder	Up to 2015	66 RCTs	Adults diagnosed with social anxiety	11,597	NR	Any medication administered to treat social anxiety versus an active or nonactive placebo	Trials that included only a subset of participants that met the review inclusion criteria in the analysis	Treatment efficacy measured as clinical global impressions and relapse rate, and treatment tolerability	Cochrane	The quality of evidence of efficacy for SSRIs is low to moderate. The tolerability was lower than placebo.
Sugarman, 2017 (36)	Antidepressants in obsessive-compulsive disorders	1994-2008	56 RCTs	DSM-IV-based anxiety disorders	15,167	NR	Second generation antidepressant for anxiety-related psychiatric diagnoses	Not second generation antidepressant	Changes in pre-post scores on symptom inventories	NR	Overall score changes were smaller for OCD compared to other anxiety disorders for both antidepressants and placebo.
Yee, 2018 (37)	Vortioxetine	Up to 2017	7 RCTs	Patients* in treatment for anxiety disorders	2,391	NR	Vortioxetine for treating anxiety disorders	Not human studies and not English language	Change from baseline at the final week of study on the Hamilton Anxiety Scale	NR	The evidence supports the use of vortioxetine for anxiety disorders. However, further long-term placebo-control observational studies or a postmarket survey would strengthen the existing evidence.
**Multimodal combined treatment comparisons**											
Bandelow, 2015 (15)	Efficacy of all treatments for anxiety disorders	1980-2013	234 RCTs	Adults with DSM-based GAD, panic disorder or social anxiety	37,333	NR	Effective drugs, psychological therapies and combined treatments, as shown in RCTs	Missing information, sample size of less than 10, children and adolescents	Evaluation of pre-post effect sizes for treatments	SIGN	The average pre-post effect sizes of medications were more effective than psychotherapies. Psychotherapy effects did not differ from pill placebos.
Ho, 2016 (38)	Stepped care prevention and treatment compared with care-as-usual	Up to 2015	10 RCTs	Participants with depressive and/or anxiety disorders	488	63.5%	Stepped care treatment or prevention (versus care-as-usual or wait-list)	Studies with no “stepping-up” criteria	Changes in pre-post scores on symptom inventories	Cochrane	Stepped-care model appeared to be better than care-as-usual in treating anxiety disorders.
Bandelow, 2018 (14)	Enduring effects of treatments for anxiety disorders	1980-2016	93 RCTs	Adults with DSM-based GAD, panic disorder or social anxiety	NR	NR	Effective drugs, psychological therapies and combined treatments (RCTs with up to 24 months follow-up)	Missing information, sample size of less than 10, children and adolescents	Evaluation of effect sizes in different follow-up moments	SIGN	Not only psychotherapy but also medications and, to a lesser extent, placebo conditions have enduring effects. Long-lasting treatment effects observed in the follow-up period may be superimposed.

**Footnotes**: **CCDANCTR**: The Cochrane Depression, Anxiety and Neurosis Review Group’s Specialized Register; **CDSR**: Cochrane Database of Systematic Reviews; **CENTRAL**: The Cochrane Central Register of Controlled Trials; **CINAHL**: Cumulative Index to Nursing and Allied Health Literature; **Cochrane**: Cochrane’s Collaboration Tool to Assess Risk of Bias; **CRD**: Centre for Reviews and Dissemination; **DAI**: Dissertation Abstracts International; **ICTRP**: World Health Organization’s trials portal; **PBSC**: Psychology and Behavioral Sciences Collection; **SIGN**: Scottish Intercollegiate Guidelines Network.

* Includes nonadult participants; **CBT**: cognitive behavioral therapy; **GAD**: generalized anxiety disorders; **PTSD**: posttraumatic stress disorders; **RCT**: randomized controlled trials; **NR**: data not reported, not available or not comprehensively summarized; **DSM**: Diagnostic and Statistical Manual; **SSRI**: selective serotonin reuptake inhibitors; **OCD:** obsessive compulsive disorder.

**Table 2:** Assessment of the quality and risk of bias of 19 selected systematic reviews of treatments for anxiety disorders, in accordance with the A MeaSurement Tool to Assess systematic Reviews (AMSTAR 2.0) and Risk Of Bias In Systematic reviews (ROBIS).

	1	2	3	4	5	6	7	8	9	10	11	12	13	14	15	16	AMSTAR	ROBIS
Author	PICO	Protocol	Study selection	Literature search	Selection in duplicate	Extraction in duplicate	Excluded studies	Included studies	Individual risk of bias	Funding of studies	Appropriate meta-analysis	Impact of risk of bias	Interpreting/ discussing results	Discussion of heterogeneity	Publication bias	Conflict of interest	Quality	Risk of bias
**Nonbiological or psychological treatments**																		
Mayo-Wilson, 2013 (25)	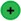 [Table-fn TFN01t02]	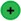 [Table-fn TFN01t02]	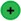 [Table-fn TFN01t02]	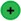 [Table-fn TFN01t02]	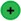 [Table-fn TFN01t02]	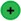 [Table-fn TFN01t02]	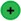 [Table-fn TFN01t02]	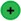 [Table-fn TFN01t02]	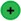 [Table-fn TFN01t02]	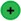 [Table-fn TFN01t02]	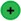 [Table-fn TFN01t02]	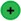 [Table-fn TFN01t02]	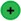 [Table-fn TFN01t02]	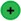 [Table-fn TFN01t02]	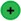 [Table-fn TFN01t02]	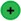 [Table-fn TFN01t02]	High	Low
Jayakody, 2014 (22)	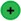 [Table-fn TFN01t02]	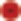 [Table-fn TFN02t02]	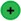 [Table-fn TFN01t02]	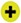 [Table-fn TFN03t02]	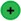 [Table-fn TFN01t02]	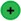 [Table-fn TFN01t02]	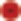 [Table-fn TFN02t02]	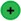 [Table-fn TFN01t02]	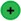 [Table-fn TFN01t02]	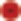 [Table-fn TFN02t02]	NA	NA	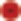 [Table-fn TFN02t02]	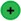 [Table-fn TFN01t02]	NA	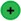 [Table-fn TFN01t02]	Low	Uncertain
Arnberg, 2014 (26)	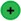 [Table-fn TFN01t02]	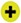 [Table-fn TFN03t02]	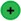 [Table-fn TFN01t02]	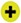 [Table-fn TFN03t02]	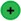 [Table-fn TFN01t02]	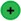 [Table-fn TFN01t02]	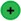 [Table-fn TFN01t02]	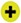 [Table-fn TFN03t02]	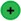 [Table-fn TFN01t02]	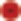 [Table-fn TFN02t02]	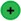 [Table-fn TFN01t02]	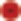 [Table-fn TFN02t02]	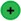 [Table-fn TFN01t02]	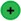 [Table-fn TFN01t02]	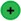 [Table-fn TFN01t02]	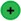 [Table-fn TFN01t02]	Moderate	Low
Abbass, 2014 (27)	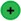 [Table-fn TFN01t02]	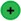 [Table-fn TFN01t02]	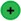 [Table-fn TFN01t02]	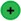 [Table-fn TFN01t02]	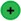 [Table-fn TFN01t02]	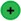 [Table-fn TFN01t02]	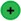 [Table-fn TFN01t02]	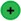 [Table-fn TFN01t02]	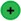 [Table-fn TFN01t02]	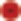 [Table-fn TFN02t02]	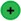 [Table-fn TFN01t02]	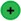 [Table-fn TFN01t02]	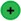 [Table-fn TFN01t02]	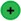 [Table-fn TFN01t02]	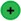 [Table-fn TFN01t02]	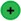 [Table-fn TFN01t02]	High	Low
Norton, 2015 (23)	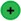 [Table-fn TFN01t02]	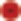 [Table-fn TFN02t02]	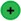 [Table-fn TFN01t02]	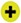 [Table-fn TFN03t02]	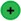 [Table-fn TFN01t02]	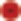 [Table-fn TFN02t02]	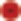 [Table-fn TFN02t02]	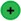 [Table-fn TFN01t02]	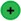 [Table-fn TFN01t02]	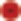 [Table-fn TFN02t02]	NA	NA	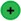 [Table-fn TFN01t02]	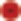 [Table-fn TFN02t02]	NA	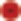 [Table-fn TFN02t02]	Moderate	Uncertain
Olthuis, 2015 (28)	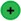 [Table-fn TFN01t02]	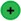 [Table-fn TFN01t02]	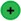 [Table-fn TFN01t02]	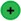 [Table-fn TFN01t02]	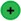 [Table-fn TFN01t02]	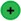 [Table-fn TFN01t02]	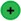 [Table-fn TFN01t02]	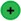 [Table-fn TFN01t02]	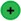 [Table-fn TFN01t02]	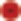 [Table-fn TFN02t02]	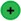 [Table-fn TFN01t02]	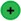 [Table-fn TFN01t02]	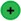 [Table-fn TFN01t02]	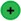 [Table-fn TFN01t02]	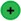 [Table-fn TFN01t02]	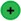 [Table-fn TFN01t02]	High	Low
Wu, 2015 (30)	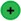 [Table-fn TFN01t02]	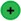 [Table-fn TFN01t02]	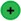 [Table-fn TFN01t02]	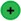 [Table-fn TFN01t02]	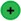 [Table-fn TFN01t02]	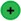 [Table-fn TFN01t02]	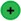 [Table-fn TFN01t02]	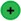 [Table-fn TFN01t02]	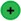 [Table-fn TFN01t02]	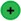 [Table-fn TFN01t02]	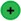 [Table-fn TFN01t02]	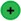 [Table-fn TFN01t02]	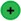 [Table-fn TFN01t02]	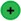 [Table-fn TFN01t02]	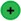 [Table-fn TFN01t02]	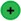 [Table-fn TFN01t02]	High	Low
Newby, 2015 (29)	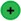 [Table-fn TFN01t02]	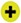 [Table-fn TFN03t02]	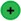 [Table-fn TFN01t02]	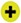 [Table-fn TFN03t02]	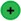 [Table-fn TFN01t02]	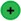 [Table-fn TFN01t02]	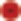 [Table-fn TFN02t02]	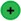 [Table-fn TFN01t02]	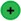 RCT 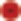 NRCT	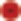 [Table-fn TFN02t02]	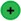 [Table-fn TFN01t02]	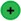 [Table-fn TFN01t02]	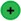 [Table-fn TFN01t02]	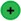 [Table-fn TFN01t02]	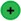 [Table-fn TFN01t02]	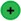 [Table-fn TFN01t02]	Low	Uncertain
Piccirillo, 2016 (24)	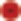 [Table-fn TFN02t02]	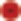 [Table-fn TFN02t02]	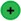 [Table-fn TFN01t02]	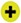 [Table-fn TFN03t02]	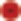 [Table-fn TFN02t02]	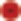 [Table-fn TFN02t02]	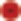 [Table-fn TFN02t02]	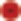 [Table-fn TFN02t02]	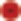 [Table-fn TFN02t02]	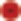 [Table-fn TFN02t02]	NA	NA	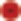 [Table-fn TFN02t02]	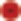 [Table-fn TFN02t02]	NA	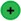 [Table-fn TFN01t02]	Critical low	High
Stubbs, 2017 (31)	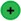 [Table-fn TFN01t02]	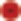 [Table-fn TFN02t02]	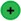 [Table-fn TFN01t02]	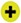 [Table-fn TFN03t02]	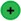 [Table-fn TFN01t02]	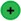 [Table-fn TFN01t02]	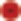 [Table-fn TFN02t02]	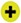 [Table-fn TFN03t02]	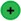 [Table-fn TFN01t02]	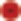 [Table-fn TFN02t02]	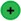 [Table-fn TFN01t02]	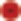 [Table-fn TFN02t02]	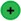 [Table-fn TFN01t02]	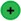 [Table-fn TFN01t02]	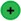 [Table-fn TFN01t02]	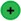 [Table-fn TFN01t02]	Moderate	Low
Cramer, 2018 (32)	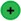 [Table-fn TFN01t02]	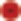 [Table-fn TFN02t02]	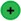 [Table-fn TFN01t02]	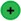 [Table-fn TFN01t02]	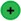 [Table-fn TFN01t02]	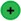 [Table-fn TFN01t02]	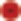 [Table-fn TFN02t02]	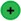 [Table-fn TFN01t02]	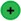 [Table-fn TFN01t02]	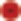 [Table-fn TFN02t02]	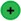 [Table-fn TFN01t02]	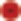 [Table-fn TFN02t02]	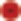 [Table-fn TFN02t02]	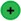 [Table-fn TFN01t02]	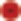 [Table-fn TFN02t02]	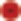 [Table-fn TFN02t02]	Low	Uncertain
**Biological or pharmacological treatments**																		
Li, 2014 (33)	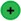 [Table-fn TFN01t02]	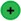 [Table-fn TFN01t02]	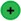 [Table-fn TFN01t02]	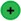 [Table-fn TFN01t02]	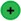 [Table-fn TFN01t02]	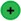 [Table-fn TFN01t02]	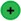 [Table-fn TFN01t02]	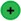 [Table-fn TFN01t02]	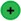 [Table-fn TFN01t02]	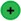 [Table-fn TFN01t02]	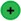 [Table-fn TFN01t02]	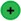 [Table-fn TFN01t02]	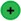 [Table-fn TFN01t02]	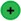 [Table-fn TFN01t02]	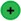 [Table-fn TFN01t02]	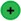 [Table-fn TFN01t02]	High	Low
Patterson, 2016 (34)	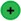 [Table-fn TFN01t02]	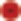 [Table-fn TFN02t02]	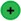 [Table-fn TFN01t02]	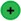 [Table-fn TFN01t02]	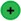 [Table-fn TFN01t02]	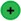 [Table-fn TFN01t02]	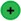 [Table-fn TFN01t02]	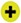 [Table-fn TFN03t02]	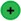 [Table-fn TFN01t02]	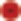 [Table-fn TFN02t02]	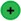 [Table-fn TFN01t02]	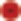 [Table-fn TFN02t02]	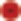 [Table-fn TFN02t02]	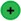 [Table-fn TFN01t02]	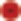 [Table-fn TFN02t02]	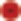 [Table-fn TFN02t02]	Low	Uncertain
Williams, 2017 (35)	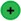 [Table-fn TFN01t02]	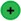 [Table-fn TFN01t02]	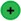 [Table-fn TFN01t02]	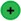 [Table-fn TFN01t02]	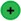 [Table-fn TFN01t02]	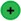 [Table-fn TFN01t02]	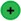 [Table-fn TFN01t02]	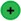 [Table-fn TFN01t02]	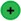 [Table-fn TFN01t02]	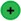 [Table-fn TFN01t02]	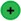 [Table-fn TFN01t02]	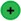 [Table-fn TFN01t02]	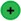 [Table-fn TFN01t02]	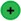 [Table-fn TFN01t02]	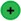 [Table-fn TFN01t02]	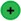 [Table-fn TFN01t02]	High	Low
Sugarman, 2017 (36)	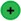 [Table-fn TFN01t02]	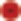 [Table-fn TFN02t02]	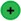 [Table-fn TFN01t02]	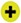 [Table-fn TFN03t02]	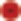 [Table-fn TFN02t02]	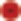 [Table-fn TFN02t02]	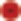 [Table-fn TFN02t02]	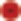 [Table-fn TFN02t02]	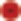 [Table-fn TFN02t02]	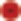 [Table-fn TFN02t02]	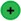 [Table-fn TFN01t02]	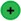 [Table-fn TFN01t02]	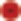 [Table-fn TFN02t02]	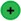 [Table-fn TFN01t02]	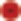 [Table-fn TFN02t02]	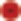 [Table-fn TFN02t02]	Critical low	High
Yee, 2018 (37)	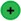 [Table-fn TFN01t02]	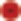 [Table-fn TFN02t02]	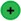 [Table-fn TFN01t02]	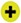 [Table-fn TFN03t02]	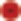 [Table-fn TFN02t02]	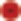 [Table-fn TFN02t02]	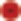 [Table-fn TFN02t02]	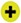 [Table-fn TFN03t02]	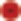 [Table-fn TFN02t02]	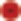 [Table-fn TFN02t02]	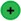 [Table-fn TFN01t02]	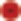 [Table-fn TFN02t02]	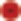 [Table-fn TFN02t02]	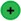 [Table-fn TFN01t02]	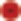 [Table-fn TFN02t02]	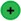 [Table-fn TFN01t02]	Critical low	High
**Multimodal combinedtreatment comparisons**																		
Bandelow, 2015 (15)	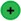 [Table-fn TFN01t02]	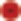 [Table-fn TFN02t02]	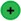 [Table-fn TFN01t02]	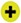 [Table-fn TFN03t02]	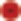 [Table-fn TFN02t02]	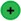 [Table-fn TFN01t02]	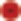 [Table-fn TFN02t02]	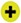 [Table-fn TFN03t02]	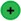 [Table-fn TFN01t02]	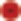 [Table-fn TFN02t02]	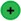 [Table-fn TFN01t02]	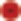 [Table-fn TFN02t02]	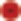 [Table-fn TFN02t02]	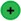 [Table-fn TFN01t02]	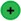 [Table-fn TFN01t02]	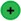 [Table-fn TFN01t02]	Low	Uncertain
Ho, 2016 (38)	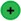 [Table-fn TFN01t02]	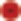 [Table-fn TFN02t02]	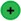 [Table-fn TFN01t02]	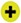 [Table-fn TFN03t02]	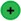 [Table-fn TFN01t02]	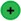 [Table-fn TFN01t02]	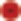 [Table-fn TFN02t02]	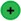 [Table-fn TFN01t02]	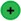 [Table-fn TFN01t02]	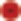 [Table-fn TFN02t02]	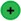 [Table-fn TFN01t02]	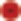 [Table-fn TFN02t02]	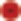 [Table-fn TFN02t02]	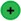 [Table-fn TFN01t02]	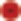 [Table-fn TFN02t02]	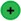 [Table-fn TFN01t02]	Low	Uncertain
Bandelow, 2018 (14)	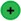 [Table-fn TFN01t02]	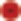 [Table-fn TFN02t02]	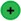 [Table-fn TFN01t02]	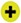 [Table-fn TFN03t02]	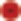 [Table-fn TFN02t02]	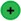 [Table-fn TFN01t02]	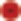 [Table-fn TFN02t02]	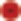 [Table-fn TFN02t02]	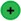 [Table-fn TFN01t02]	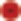 [Table-fn TFN02t02]	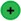 [Table-fn TFN01t02]	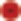 [Table-fn TFN02t02]	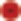 [Table-fn TFN02t02]	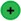 [Table-fn TFN01t02]	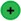 [Table-fn TFN01t02]	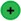 [Table-fn TFN01t02]	Low	Uncertain

Footnotes:

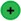
Yes

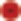
No

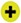
Partial Yes

**NA:** not applicable - no meta-analysis.

**RCT/NRCT**: randomized controlled trials/nonrandomized controlled trials.

**Supplementary Table 2:** List of excluded studies.

Author, Year	Reason for exclusion
Alladin A., 2014	Not a systematic review
Bluett E., 2014	Not a systematic review
Palm U., 2017	Not a systematic review
Spiegel S., 2014	Not a systematic review
Reinhold J., 2015	Not a systematic review
Shahar B., 2014	Not a systematic review
Gotink R., 2015	No specific recent data

**REFERENCES** 1. Alladin A. The wounded self: new approach to understanding and treating anxiety disorders. Am J Clin Hypn. 2014;56(4):368-88. 2. Bluett EJ, Homan KJ, Morrison KL, Levin ME, Twohig MP. Acceptance and commitment therapy for anxiety and OCD spectrum disorders: an empirical review. J Anxiety Disord. 2014;28(6):612-24. 3. Palm U, Leitner B, Kirsch B, Behler N, Kumpf U, Wulf L, et al. Prefrontal tDCS and sertraline in obsessive compulsive disorder: a case report and review of the literature. Neurocase. 2017;23(2):173-7. 4. Spiegel SB. Current issues in the treatment of specific phobia: recommendations for innovative applications of hypnosis. Am J Clin Hypn. 2014;56(4):389-404. 5. Reinhold JA, Rickels K. Pharmacological treatment for generalized anxiety disorder in adults: an update. Expert Opin Pharmacother. 2015;16(11):1669-81. 6. Shahar B. Emotion-focused therapy for the treatment of social anxiety: an overview of the model and a case description. Clin Psychol Psychother. 2014;21(6):536-47. 7. Gotink RA, Chu P, Busschbach JJ, Benson H, Fricchione GL, Hunink MG. Standardised mindfulness-based interventions in healthcare: an overview of systematic reviews and meta-analyses of RCTs. PLoS One. 2015;10(4):e0124344.

**Supplementary Table 3:** Ratings of Phase 2 and Phase 3 of ROBIS (Risk Of Bias In Systematic review) in 19 selected systematic reviews on the treatment of anxiety disorders (2013-2018).

Author	Phase 2				Phase 3			ROBIS rating
	1. Study eligibility criteria	2. Identification and selection	3. Data collection and appraisal	4. Synthesis and findings	A. Interpretation of concerns (Phase 2 assessment)?	B. Relevance of identified studies?	C. Avoid emphasizing results?	
**Nonbiological or psychological treatments**								
Mayo-Wilson, 2013 (25)	Low	Low	Low	Low	Yes	Yes	Yes	Low risk
Jayakody, 2014 (22)	Low	Low	Low	High	Yes	Probably Yes	Yes	Uncertain
Arnberg, 2014 (26)	Low	Low	Low	Low	Yes	Probably Yes	Yes	Low risk
Abbass, 2014 (27)	Low	Low	Low	Low	Yes	Yes	Yes	Low risk
Norton, 2015 (23)	Low	Low	Low	High	Yes	Probably Yes	Probably Yes	Uncertain
Olthuis, 2015 (28)	Low	Low	Low	Low	Yes	Yes	Yes	Low risk
Wu, 2015 (30)	Low	Low	Low	Low	Yes	Yes	Yes	Low risk
Newby, 2015 (29)	Low	Low	High	Low	Unclear	Yes	Yes	Uncertain
Piccirillo, 2016 (24)	High	High	High	High	No	Probably Yes	Unclear	High risk
Stubbs, 2017 (31)	Low	Low	Low	Low	Yes	Yes	Yes	Low risk
Cramer, 2018 (32)	Low	Low	Low	High	No	Probably Yes	Yes	Uncertain
**Biological or pharmacological treatments**								
Li, 2014 (33)	Low	Low	Low	Low	Yes	Yes	Yes	Low risk
Patterson, 2016 (34)	Low	Low	Low	High	No	Probably Yes	Probably Yes	Uncertain
Williams, 2017 (35)	Low	Low	Low	Low	Yes	Yes	Yes	Low risk
Sugarman, 2017 (36)	High	High	High	High	No	Probably Yes	Yes	High risk
Yee, 2018 (37)	High	High	High	High	No	Probably Yes	Probably Yes	High risk
**Multimodal combined treatment comparisons**								
Bandelow, 2015 (15)	Low	Unclear	Low	Unclear	Unclear	Probably Yes	Yes	Uncertain
Ho, 2016 (38)	Low	Low	Low	High	No	Yes	Probably Yes	Uncertain
Bandelow, 2018 (14)	Low	Unclear	Low	Unclear	Unclear	Probably Yes	Yes	Uncertain
